# Expression of androgen receptor in non-muscle-invasive bladder cancer predicts the preventive effect of androgen deprivation therapy on tumor recurrence

**DOI:** 10.18632/oncotarget.7358

**Published:** 2016-02-12

**Authors:** Koji Izumi, Yusuke Ito, Hiroshi Miyamoto, Yasuhide Miyoshi, Junichi Ota, Masatoshi Moriyama, Tetsuo Murai, Hiroyuki Hayashi, Yoshiaki Inayama, Kenichi Ohashi, Masahiro Yao, Hiroji Uemura

**Affiliations:** ^1^ Department of Urology, Yokohama City University Graduate School of Medicine, Yokohama, Japan; ^2^ Department of Urology, Yokohama Municipal Citizen's Hospital, Yokohama, Japan; ^3^ Departments of Pathology and Urology, Johns Hopkins University School of Medicine, Baltimore, MD, USA; ^4^ Department of Urology, Yokohama City University Medical Center, Yokohama, Japan; ^5^ Department of Urology, International Goodwill Hospital, Yokohama, Japan; ^6^ Department of Pathology, Yokohama Municipal Citizen's Hospital, Yokohama, Japan; ^7^ Department of Pathology, Yokohama City University Medical Center, Yokohama, Japan; ^8^ Department of Pathology, Yokohama City University Graduate School of Medicine, Yokohama, Japan

**Keywords:** bladder cancer, recurrence, androgen receptor, androgen deprivation therapy

## Abstract

Our recent retrospective study revealed a significantly reduced risk of bladder cancer (BC) recurrence in men who received androgen deprivation therapy (ADT) for their prostate cancer. However, whether androgen receptor (AR) signals contributed to the preventive effect of ADT remained unclear because ADT could reduce serum estrogens as well. The purpose of this study is to investigate the associations between the expression of AR/estrogen receptors (ERs) and BC recurrence in patients treated with ADT. We immunohistochemically stained 72 BCs and 42 corresponding normal urothelial tissues. AR/ERα/ERβ were positive in 44(61%)/22(31%)/39(54%) tumors and 35(83%)/24(57%)/34(81%) corresponding normal urothelial tissues, respectively. There were no statistically significant correlations between AR/ERα/ERβ expression and clinicopathological features of BC. With a median follow-up of 31.3 months, 12 (43%) of 28 patients with AR-negative tumor versus 11 (23%) of 44 patients with AR-positive tumor experienced BC recurrence. Thus, patients with AR-positive tumor had a significantly lower risk of BC recurrence (P=0.031), compared with those with AR-negative tumor. Meanwhile, the expression of ERα/ERβ in tumors and that of AR/ERα/ERβ in normal urothelial tissues were not significantly correlated with BC recurrence. A multivariate analysis revealed AR positivity in tumors as an independent prognosticator (hazard ratio: 0.27; 95% confidence interval: 0.11-0.67) for BC recurrence. These results indicate that ADT prevents BC recurrence via the AR pathway, but not via the ERα/ERβ pathways.

## INTRODUCTION

Men have 3-4 times higher incidence of bladder cancer (BC) than women [1]. Although approximately 80% of patients are present with non-muscle-invasive BC (NMIBC) at the initial diagnosis, 36-51% of them recur despite of currently available adjuvant instillation therapy, such as intravesical instillation of an anthracycline or bacillus Calmette-Guérin (BCG), and approximately 10% of them eventually progressed to muscle invasion [2]. In addition, developments in treatment modalities for BC did not result in an improvement in disease mortality rates for several decades [3]. Therefore, new treatment is urgently needed to prevent both recurrence and progression of BC.

Using preclinical models, we and others have revealed the involvement of androgen receptor (AR) signaling in BC development [4–6]. However, prognostic significance of AR expression in BC recurrence is still controversial [7–11]. Moreover, there is no significant gender-specific difference in the expression levels of AR [7], suggesting that circulating serum androgens may be critical for AR-stimulated BC development.

Recently, we demonstrated first clinical evidence indicating that androgen deprivation therapy (ADT) prevented BC recurrence [12]. In brief, in patients with double primary cancers of the prostate and bladder, ADT for their prostate cancer, mostly with a LHRH analogue, significantly reduced the risk of BC recurrence [hazard ratio (HR): 0.29]. LHRH analogues suppress secretion of follicle-stimulating hormone (FSH) and luteinizing hormone (LH) from the pituitary gland resulting in suppression of androgens from the testis. Since estrogens in men are mainly produced by aromatization of testosterone, LHRH analogue deprives estrogens as well [13]. Estrogens-mediated estrogen receptor (ER) signals have also been implicated in BC development [14–16]. Even in men, estrogens whose serum levels are similar to those in postmenopausal women have physiological functions, such as bone health, regulation of body fat, and sexual function [17].

Thus, whether AR signals were responsible for the preventive effect of ADT on BC recurrence was still open question. The purpose of this study is to investigate the associations between AR/ERα/ERβ expression in surgical specimens and BC recurrence in patients treated with ADT.

## RESULTS

### AR/ERα/ERβ expression in tumors and corresponding normal tissues

**Figure 1:**
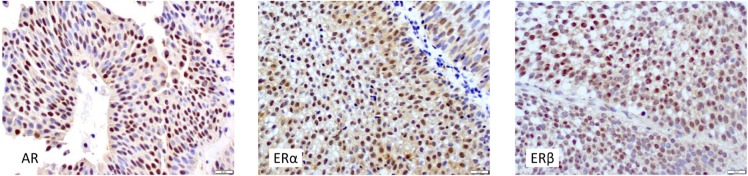
Immunohistochemistry of AR, ERα, and ERβ in urothelial tumors Original magnification: x400.

We immunohistochemically evaluated 72 BCs and 42 corresponding normal tissues for the expression of AR, ERα, and ERβ. Positive signals for all three receptors were detected predominantly in the nuclei of neoplastic (Figure 1) or non-neoplastic (figure not shown) urothelial cells. AR/ERα/ERβ were positive in 44(61%)/22(31%)/39(54%) tumors and 35(83%)/24(57%)/34(81%) corresponding normal-appearing urothelial tissues, respectively. In accordance with our previous findings [7], AR (*P* = 0.020)/ERα (*P* = 0.006)/ERβ (*P* = 0.005) expression was significantly lower in tumors than in non-neoplastic urothelial tissues.

### Patient characteristics

AR/ERα/ERβ expression in BCs and corresponding normal tissues, in relation to patient/tumor characteristics, is shown in Tables 1 and 2. There were no statistically significant correlations between each receptor expression in tumors or normal tissues and clinicopatholgical features of BCs.

**Table 1:** Correlations between AR/ERα/ERβ Expression in Tumors and Clinicopathological Features of BC

Characteristics	AR(−), n (%)	AR(+), n (%)	*P*	ERα(−), n (%)	ERα(+), n (%)	*P*	ERβ(−), n (%)	ERβ(+), n (%)	*P*
No.	28 (38.9)	44 (61.1)		50 (69.4)	22 (30.6)		33 (45.8)	39 (54.2)	
Age, y [median (IQR)]	74.1 (68.5-76.5)	70.8 (63.5-75.1)	0.494	72.9 (64.7-80.4)	71.1 (63.5-80.1)	0.755	74.2 (64.7-77.2)	70.7 (63.5-75.0)	0.203
Tumor grade			0.867			0.436			0.691
1	6 (21.4)	12 (27.3)		11 (22.0)	7 (31.8)		7 (21.2)	11 (28.2)	
2	14 (50.0)	18 (40.9)		21 (42.0)	11 (50.0)		17 (51.5)	15 (38.5)	
3	7 (25.0)	11 (25.0)		14 (28.0)	4 (18.2)		8 (24.2)	10 (25.6)	
Not applicable	1 (3.6)	3 (6.8)		4 (8.0)	0 (0)		1 (3.0)	3 (7.7)	
Pathological T stage			0.555			0.477			0.762
pTa	23 (82.1)	31 (70.5)		36 (72.0)	18 (81.8)		26 (78.8)	28 (71.8)	
pT1	4 (14.3)	10 (22.7)		10 (20.0)	4 (18.2)		6 (18.2)	8 (20.5)	
pTis	1 (3.6)	3 (6.8)		4 (8.0)	0 (0)		1 (3.0)	3 (7.7)	
Tumor size			0.252			1.000			0.257
<3 cm	24 (85.7)	32 (72.7)		39 (78.0)	17 (77.3)		28 (84.8)	28 (71.8)	
≥3 cm	4 (14.3)	12 (27.3)		11 (22.0)	5 (22.7)		5 (15.2)	11 (28.2)	
Tumor number			0.809			0.443			0.637
Single	15 (53.6)	21 (47.7)		27 (54.0)	9 (40.9)		18 (54.5)	18 (46.2)	
Multiple	13 (46.4)	23 (52.3)		23 (46.0)	13 (59.1)		15 (45.5)	21 (53.8)	
Concomitant CIS			0.471			0.421			0.275
No	26 (92.9)	38 (86.4)		43 (86.0)	21 (95.5)		31 (93.9)	33 (84.6)	
Yes	2 (7.1)	6 (13.6)		7 (14.0)	1 (4.5)		2 (6.1)	6 (15.4)	
Intravesical instillation			0.352			0.747			1.000
No	16 (57.1)	20 (45.5)		24 (48.0)	12 (54.5)		17 (51.5)	19 (48.7)	
Anthracycline	9 (32.1)	13 (29.5)		15 (30.0)	7 (31.8)		10 (30.3)	12 (30.8)	
BCG	3 (10.7)	11 (25.0)		11 (22.0)	3 (13.6)		6 (18.2)	8 (20.5)	

Abbreviations: CIS, carcinoma in situ; BCG, Bacillus Calmette–Guérin.

**Table 2:** Correlations between AR/ERα/ERβ Expression in Normal Urothelial Tissues and Clinicopathological Features of BC

Characteristics	AR(−), n (%)	AR(+), n (%)	*P*	ERα(−), n (%)	ERα(+), n (%)	*P*	ERβ(−), n (%)	ERβ(+), n (%)	*P*
No.	7(16.7)	35 (83.3)		18 (42.9)	24 (57.1)		8 (19.0)	34 (81.0)	
Age, y [median (IQR)]	77.2 (75.8-80.1)	77.6 (73.1-80.2)	0.644	75.5 (73.0-78.1)	78.9 (74.6-83.6)	0.091	77.2 (74.2-78.4)	77.2 (73.9-80.5)	0.604
Tumor grade			0.222			0.845			0.101
1	1 (14.3)	12 (34.3)		6 (33.3)	7 (29.2)		0 (0)	13 (38.2)	
2	6 (85.7)	13 (37.1)		9 (50.0)	10 (41.7)		5 (62.5)	14 (41.2)	
3	0 (0)	6 (17.1)		2 (11.1)	4 (16.7)		2 (25.0)	4 (11.8)	
Not applicable	0 (0)	4 (11.4)		1 (5.6)	3 (12.5)		1 (12.5)	3 (8.8)	
Pathological T stage			1.000			0.861			0.275
pTa	6 (85.7)	27 (77.1)		15 (83.3)	18 (75.0)		5 (62.5)	28 (82.4)	
pT1	1 (14.3)	4 (11.4)		2 (11.1)	3 (12.5)		2 (25.0)	3 (8.8)	
pTis	0 (0)	4 (11.4)		1 (5.6)	3 (12.5)		1 (12.5)	3 (8.8)	
Tumor size			0.631			1.000			0.168
<3 cm	5 (71.4)	28 (80.0)		14 (77.8)	19 (79.2)		8 (100)	25 (73.5)	
≥3 cm	2 (28.6)	7 (20.0)		4 (22.2)	5 (20.8)		0 (0)	9 (26.5)	
Tumor number			1.000			0.353			1.000
Single	4 (57.1)	21 (60.0)		12 (66.7)	12 (50.0)		5 (62.5)	19 (55.9)	
Multiple	3 (42.9)	14 (40.0)		6 (33.3)	12 (50.0)		3 (37.5)	15 (44.1)	
Concomitant CIS			0.532			1.000			1.000
No	6 (85.7)	32 (91.4)		16 (88.9)	22 (91.7)		7 (87.5)	31 (91.2)	
Yes	1 (14.3)	3 (8.6)		2 (11.1)	2 (8.3)		1 (12.5)	3 (8.8)	
Intravesical instillation			0.644			0.919			0.746
No	3 (42.9)	15 (42.9)		8 (44.4)	10 (41.7)		3 (37.5)	15 (44.1)	
Anthracycline	4 (57.1)	14 (40.0)		7 (38.9)	11 (45.8)		3 (37.5)	15 (44.1)	
BCG	0 (0)	6 (17.1)		3 (16.7)	3 (12.5)		2 (25.0)	4 (11.8)	

Abbreviations: CIS, carcinoma in situ; BCG, Bacillus Calmette–Guérin.

### Expression of AR, ERα, and ERβ in BCs and tumor recurrence

**Figure 2:**
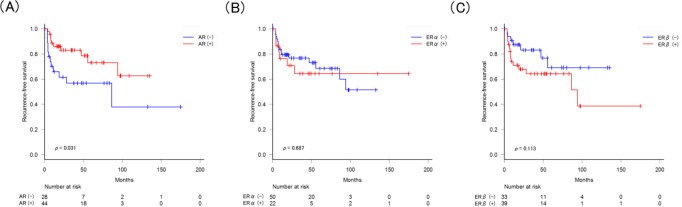
Recurrence-free survival according to the expression of AR/ERα/ERβ in tumors

We next performed Kaplan-Meier analyses coupled with log-rank tests to assess possible associations between staining of AR, ERα, and ERβ in BCs and tumor recurrence. With a median follow-up of 31.3 months, 12 (43%) of 28 patients with AR-negative tumor *versus* 11 (23%) of 44 patients with AR-positive tumor experienced BC recurrence. Median time to recurrence was 8.2 (interquartile range [IQR]: 4.7-20.4) months. Thus, patients with AR-positive tumor had a significantly lower risk of BC recurrence (5-year actuarial recurrence-free survival: 73% v 57%; *P* = 0.031, Figure 2A), compared with those with AR-negative tumor. On the other hand, ERα (Figure 2B) or ERβ (Figure 2C) positivity in tumors did not significantly correlate with tumor recurrence.

To see whether AR expression was an independent predictor of recurrence, multivariate analysis was performed with Cox model, including AR positivity and other well-known clinicopathological risk factors for BC recurrence (Table 3). Of these, AR positivity (HR: 0.27; 95% confidence interval (CI): 0.11-0.67) as well as multiple tumor (HR: 3.12; 95%CI: 1.22-7.95) was found to be an independent prognosticator of BC recurrence.

**Table 3:** Univariate and Multivariate Analyses for BC Recurrence in ADT Patients

Variables	Univariate		Multivariate	
	HR (95%CI)	*P*	HR (95%CI)	*P*
Age, continuous value	1.05 (0.98-1.13)	0.178	1.07 (0.99-1.15)	0.076
AR expression (positive vs. negative)	0.35 (0.15-0.84)	0.018	0.27 (0.11-0.67)	0.005
Tumor grade (3 vs. 1,2)	0.99 (0.36-2.72)	0.984		
Tumor stage (pT1 vs. pTa)	0.63 (0.19-2.15)	0.461		
Tumor size (≥3 cm vs. <3 cm)	1.61 (0.58-4.45)	0.359		
Tumor number (multiple vs. single)	2.05 (0.85-4.98)	0.111	3.12 (1.22-7.95)	0.017
Carcinoma in situ (yes vs. no)	1.23 (0.29-5.30)	0.782		
Intravesical instillation (yes vs. no)	0.59 (0.23-1.46)	0.252		

### Expression of AR, ERα, and ERβ in corresponding normal tissues and tumor recurrence

**Figure 3:**
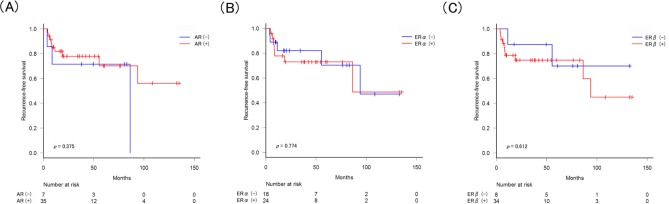
Recurrence-free survival according to the expression of AR/ERα/ERβ in normal tissues

Since de novo tumorigenesis is considered to be one of the mechanisms for BC recurrence, we then investigated associations between AR/ERα/ERβ expression in corresponding normal urothelial tissues and tumor recurrence. However, as shown in Figure 3, any of receptor expression in normal tissues did not significantly correlate with tumor recurrence.

## DISCUSSION

In the current study, we investigated very specific patients with double primary cancers of the bladder and prostate who received ADT for their prostate cancer and showed that AR positivity in BCs correlated with a reduced risk of tumor recurrence. These findings strongly support that AR signals contributed to the preventive effect of ADT on BC recurrence. On the other hand, similar to the results in patients without ADT [7], ERβ positivity in tumors showed a marginally higher risk of recurrence, suggesting that ERβ expression is not associated with the preventive effect of ADT but may be an independent prognosticator on BC recurrence. Consistent with our previous observations [7], ERα expression did not predict BC recurrence, suggesting no significant correlation between ERα signals and the ADT effect.

Although a recent review article introduced some clinical trials of new targeted therapies for BC, none of these were approved for BC treatment to date [18]. The authors assumed the lack of patient stratification by each target expression led underestimation of the efficacy of these new therapies. In our previous study [12], although we did not stratify the patients by AR expression, ADT surprisingly prevented BC recurrence with HR of 0.29. The current study then suggests that appropriate patient selection further improves the efficacy of AR inactivation in BC treatment at least for preventing recurrence of NMIBC.

For the treatment of prostate cancer, there are two major ways to target AR signals; surgical/chemical castration and anti-androgens. Castration is often associated with reduced health-related quality of life (e.g. fatigue, loss of physical capacity, emotional distress and sexual function) and bone mineral density, compared with anti-androgen monotherapy [19]. Therefore, anti-androgen monotherapy is an alternative for non-metastatic locally advanced prostate cancer, although castration with or without anti-androgen is the standard procedure. Although phase I trial targeting AR in muscle-invasive BC was initiated in the US [20], to the best of our knowledge, prospective studies for NMIBC have not yet been performed. As an adjuvant therapy, anti-androgen monotherapy might be better than castration in terms of adverse effects for the future clinical trials. Furthermore, AR knockdown in urotehlial cells by intravesical instillation of antisense oligonucleotides, as shown with those of heat shock protein 27 in BC [21], is a potential treatment option to avoid systemic adverse effects associated with ADT currently used for the treatment of prostate cancer.

Recently, some large-scale genome-wide profiling studies proposed new classifications of BC [22–25]. Interestingly, these independent studies showed significant overlaps which improved understanding of molecular features of BC [26]. Among these molecules, epidermal growth factor receptor/ERBB2 [27], Wnt/β-catenin [28], p53 [29], UDP-glucuronosyltransferase-1A [30], CD24 [31], GATA3 [32], and Slug [33] are shown to be activated or inhibited by AR signals in BC or non-neoplastic urothelial cells. In addition, recent studies have revealed the association between AR signals and Nur77 [34], ELK1 [35], or IL-8 [36] in BC. It is thus likely that AR signals contribute to modulating multiple independent pathways in BC cells.

AR expression as a predictor of better prognosis seen in the present study appears to be contrary to the tumor promoting effect of AR signals in BC [4–6]. A potential reason is that AR signals and other pathways complementarily stimulate BC development. Controversial results of an association between AR expression in BCs and their recurrence [7–11] support this hypothesis. If that is the case, when patients receive ADT, BC recurrence is induced by other pathways that are more dominant in AR-negative tumor compared with AR-positive tumor.

A recent retrospective study involving patients who received ADT for prostate cancer showed a significantly lower incidence of subsequent BC development [37]. In addition, a large scale prospective study revealed that finasteride reduced the risk of BC [38]. These clinical findings support the involvement of AR/ERα/ERβ signals in bladder tumorigenesis. Since de novo tumorigenesis might be one of the causes of BC recurrence, we also investigated associations between AR/ERα/ERβ expression in corresponding normal urothelial tissues and tumor recurrence. Since biopsy of normal urothelial tissues was not routinely performed, the sample size was much smaller than that of BC tissues. Consistent with our previous observations [7], there were no statistically significant correlations between each receptor expression in corresponding urothelial tissues and clinicopatholgical features of BC. Furthermore, any of receptor expression in corresponding urothelial tissues did not significantly correlate with tumor recurrence. This is possibly because: 1) tumor dissemination is more dominant way of recurrence; 2) adjacent urothelial tissues are no longer normal; or 3) the sample size is too small. Further investigation using larger sample size is needed to confirm the correlations between the expression of steroid hormone receptors in normal urothelium and BC recurrence.

The main limitations of the current study include its retrospective design with limited study population. However, since the incidence of primary BC in prostate cancer patients in our cohort was only 1.2% [12] and only a subset of the patients received ADT for their prostate cancer, a prospective cohort study involving men with double cancers may not be practical. Future clinical trials determining the efficacy of ADT in BC recurrence are still needed to confirm our findings.

## CONCLUSIONS

We here report the expression of AR, but not ERα or ERβ, in BC is associated with tumor recurrence in patients treated with ADT. Together with our previous observations, ADT with anti-androgen monotherapy and/or LHRH analogue is suggested to prevent BC recurrence *via* the AR pathway. In addition, AR expression in BC can be a useful marker in selecting patients for ADT.

## PATIENTS AND METHODS

### Tissue samples

We retrospectively retrieved 72 BCs and 42 corresponding normal urothelial tissues obtained by transurethral resection from patients who received ADT for their prostate cancer from 2001 to 2012 in four hospitals (Yokohama City University Hospital, Yokohama City University Medical Center, Yokohama Municipal Citizen's Hospital, and International Goodwill Hospital). Fifteen patients received LHRH monotherapy whereas others received combined androgen blockade consisting of LHRH analogue and anti-androgen. Median duration of ADT was 25.7 (IQR: 9.8-55.1) months. None of these patients received systemic chemotherapy or underwent radical cystectomy. Appropriate approval from the institutional review board at each institution was obtained before use of the tissue samples.

### Immunohistochemistry

Immunohistochemical staining was performed, as described previously [7]. Sections (4 μm thick) were immunohistochemically labeled, using a primary antibody to AR (N20 clone, dilution 1:100; Santa Cruz Biotechnology, Santa Cruz, CA, USA), ERα (E115 clone, dilution 1:100; Epitomics, Burlingame, CA, USA), and ERβ (14C8 clone, dilution 1:50; Abcam, Cambridge, MA, USA). All the stains were manually scored by one pathologist (H.M.) blinded to patient identity. The expression of each receptor was considered positive when more than 10% of urothelial tumor cells or non-neoplastic urothelial cells were immunoreactive or at least 1% of urothelial tumor cells or non-neoplastic urothelial cells showed moderate to strong intensity [7].

### Statistical analyses

The Fisher exact test was used to evaluate the association between categorized variables. Nonparametric two group comparison was carried out using Mann-Whitney U test to assess differences in continuous variables. Survival rates in patients were calculated by the Kaplan-Meier method, and comparison was made by log-rank test. The Cox proportional hazards model was used to determine statistical significance of predictors in a multivariate setting. *P* < 0.05 was considered to be statistically significant. All statistical analyses were performed with EZR (Saitama Medical Center, Jichi Medical University, Saitama, Japan), which is a graphical user interface for R (The R Foundation for Statistical Computing, Vienna, Austria). More precisely, it is a modified version of R commander designed to add statistical functions frequently used in biostatistics.
